# Towards a Better Understanding of GABAergic Remodeling in Alzheimer’s Disease

**DOI:** 10.3390/ijms18081813

**Published:** 2017-08-21

**Authors:** Karan Govindpani, Beatriz Calvo-Flores Guzmán, Chitra Vinnakota, Henry J. Waldvogel, Richard L. Faull, Andrea Kwakowsky

**Affiliations:** Centre for Brain Research, Department of Anatomy and Medical Imaging, Faculty of Medical and Health Sciences, University of Auckland, Auckland 1023, New Zealand; k.govindpani@auckland.ac.nz (K.G.); b.guzman@auckland.ac.nz (B.C.-F.G.); c.vinnakota@auckland.ac.nz (C.V.); h.waldvogel@auckland.ac.nz (H.J.W.); rlm.faull@auckland.ac.nz (R.L.F.)

**Keywords:** Alzheimer’s disease, GABA, GAD, GABA receptor, GABA transporter, GAT, E/I balance

## Abstract

γ-aminobutyric acid (GABA) is the primary inhibitory neurotransmitter in the vertebrate brain. In the past, there has been a major research drive focused on the dysfunction of the glutamatergic and cholinergic neurotransmitter systems in Alzheimer’s disease (AD). However, there is now growing evidence in support of a GABAergic contribution to the pathogenesis of this neurodegenerative disease. Previous studies paint a complex, convoluted and often inconsistent picture of AD-associated GABAergic remodeling. Given the importance of the GABAergic system in neuronal function and homeostasis, in the maintenance of the excitatory/inhibitory balance, and in the processes of learning and memory, such changes in GABAergic function could be an important factor in both early and later stages of AD pathogenesis. Given the limited scope of currently available therapies in modifying the course of the disease, a better understanding of GABAergic remodeling in AD could open up innovative and novel therapeutic opportunities.

## 1. Introduction

Alzheimer’s disease (AD) is a chronic neurodegenerative disorder, and the most prevalent form of dementia in elderly patients [1,2]. The onset of the disorder is insidious, and symptoms tend to progress gradually with age from mild memory impairment to severe dementia [3]. A range of other symptoms are associated with the progression of the disease, including the loss of language skills, cognitive deficits, loss of higher executive function, psychomotor impairment, and neuropsychiatric disturbance [3]. In advanced stages of disease progression, patients often experience significant difficulty in performing the necessary functions of daily life [3]. It has been estimated that approximately 46.8 million people were affected by dementia globally in 2015 [4]. With an estimated global incidence of approximately 9.9 million new cases per year, current trends indicate that the prevalence of dementia will double approximately once every 20 years [4]. In particular, rising life expectancies and an aging population in both developed and rapidly developing societies are mirrored by an increase in disease burden due to dementia, with dementia of the Alzheimer’s type accounting for an estimated 50–80% of the burden of dementia [4,5,6,7].

A small proportion of early-onset AD cases can be attributed to autosomal dominant mutations in one of three genes—*presenilin 1* [8], *presenilin 2* [9,10], or the amyloid precursor protein (*APP*) gene [11]. However, at present, the specific causes underlying the far more common late-onset form of the disease are poorly understood. Apart from age, several other genetic and non-genetic factors have been associated with alterations in disease risk for non-familial AD; these include gender, the apolipoprotein E genotype, baseline cognitive scores, and educational level [12]. The pathogenesis of AD is known to be associated with significant dysfunction in the cholinergic and glutamatergic neurotransmitter systems, including altered levels of these neurotransmitters and the massive degeneration of neuronal networks [13,14]. However, the progression of the disease is likely far more complex, with the involvement of additional neurotransmitter systems and molecular components. Thus, a comprehensive understanding of AD pathogenesis with a view towards the development of novel treatment strategies requires a more global consideration of neurotransmitter dysfunction in the diseased brain. γ-aminobutyric acidergic (GABAergic) dysfunction in AD has long been a neglected area of inquiry, due in no small part to the inconsistency of previous results, making this a poorly understood and controversial subject. In this review, we present and discuss evidence for GABAergic dysfunction in AD, and the role that this could play in the development of the disorder.

## 2. The γ-Aminobutyric Acid (GABA) Signaling System

**Figure 1:**
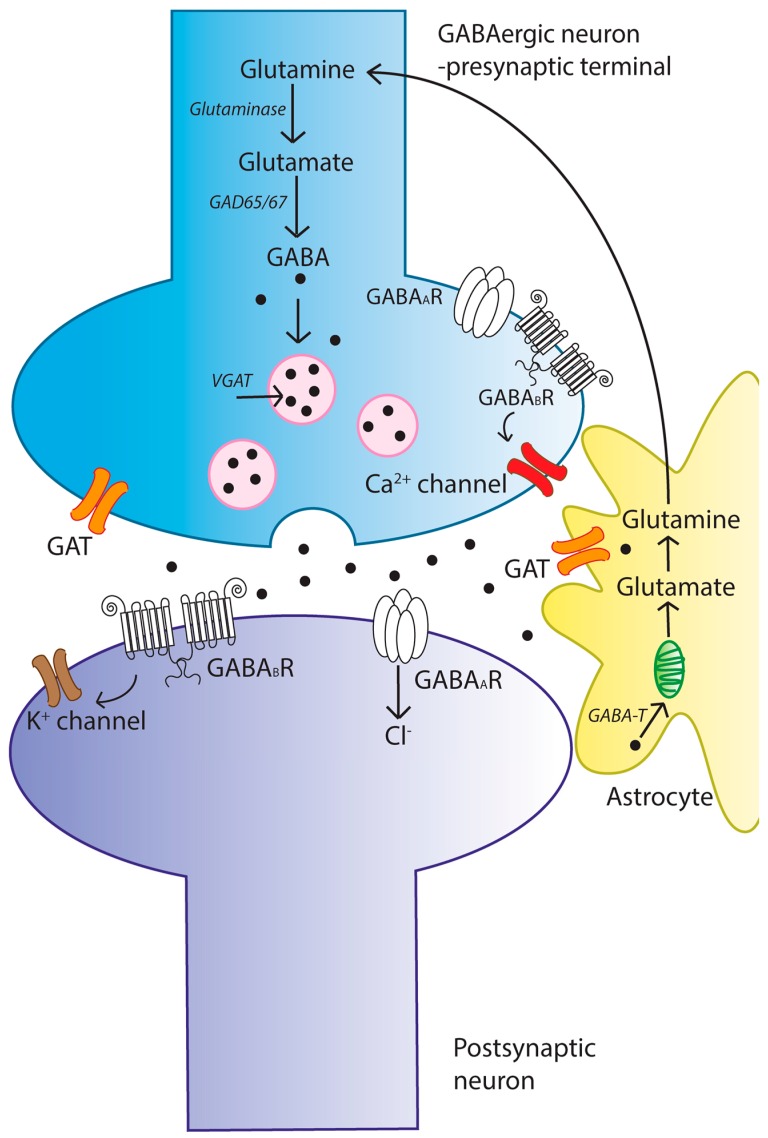
An overview of the γ-aminobutyric acid (GABA) signaling system. The schematic diagram represents a GABAergic synapse and depicts the key aspects of GABAergic signal transduction. GABA is synthesized in the pre-synaptic terminal from glutamate by glutamic acid decarboxylase (GAD). GABA is then recruited into synaptic vesicles via the action of vesicular GABA transporter (vGAT). Following membrane depolarization, GABA is released into the synapse and can bind to either ionotropic GABA_A_ receptors (GABA_A_R) or metabotropic GABA_B_ receptors (GABA_B_R) on the postsynaptic membrane, resulting in inhibition of the post-synaptic neuron. Released GABA is cleared from the synapse by membrane-bound GABA transporters (GATs), localized to neurons and astrocytes. In astrocytes, GABA is recycled into synaptic vesicles or taken up by mitochondria, where it is metabolized by GABA transaminase (GABA-T) to glutamine for neuronal uptake.

γ-aminobutyric acid (GABA) is the primary inhibitory neurotransmitter in the mammalian central nervous system (CNS), and it is crucially important in the regulation of responsiveness and excitability in human cortical networks [15,16] and in the synchronization of cortical neuronal signaling activity by networks of cortical interneurons [17,18,19]. The ubiquity of GABAergic regulation in the CNS gives this neurotransmitter a central role in a very wide range of physiological and biochemical processes—GABAergic control is involved in the regulation of cognition [20,21], memory and learning [22,23,24,25,26], motor function [27,28], circadian rhythms [29,30], neural development [31,32,33,34], adult neurogenesis [35,36,37,38], and sexual maturation [39,40,41,42]. Dysfunction in GABAergic signaling is known to be a central factor in the pathogenesis of several neurological disorders, with the role of GABA in epilepsy and the maintenance of the inhibitory-excitatory (E/I) balance in the human cortex being the subject of significant scholarship [43,44,45]. The contribution of GABAergic dysfunction to disorders such as Alzheimer’s disease (AD) [46,47], major depressive disorder [48,49], anxiety [49,50,51], autism [52,53], schizophrenia [54,55,56] and bipolar disorder [57] is also known or suspected, with many lines of evidence pointing to the underlying contribution of defects in the signaling system [53,58]. Thus, the GABAergic system has long been a major target in the development of treatment strategies for these conditions. Following is a brief description of the major components of the GABAergic system (summarized in Figure 1).

### 2.1. GABA Synthesis

GABA synthesis occurs via the α-decarboxylation of l-glutamate by the enzyme glutamic acid decarboxylase (GAD)—a single-step, irreversible reaction dependent on the availability of the cofactor pyridoxal-5′-phosphate (a vitamer of vitamin B_6_) [59,60,61]. Two major isoforms of GAD exist—a 65 kDa isoform (GAD65) and a 67 kDa isoform (GAD67); GAD65 is the major isoform of GAD expressed in the mammalian brain [62,63], localized primarily to the axon terminals of synaptosomes and interacting readily with the plasma membrane, whereas GAD67 is more widely distributed in the cytosol of cells [64,65,66,67,68,69]. The differential distribution of the two GAD isoforms corresponds well with the presence of two pools of intracellular GABA—one of which is found in vesicles and the other in the cytosol, and which are released by different mechanisms [70,71]. It has thus been suggested that GAD65, being localized in the synaptic bouton, plays a role in the synthesis of GABA released via a vesicular mechanism [72,73], whereas GAD67 likely mediates the synthesis of the cytoplasmic pool of GABA [71].

### 2.2. GABA Metabolism and Homeostasis

GABA metabolism in the brain occurs through a highly compartmentalized set of enzymatic processes. Neurotransmitter levels at GABAergic and glutamatergic synapses are largely maintained by astrocytes, through their mediation of the glutamate/GABA-glutamine cycle (summarized in Figure 1) [74]. Cortical synapses are tightly enveloped by highly specialized astroglial processes [75,76], where GABA is taken up following synaptic release [77,78,79] and catabolized to succinate in a two-step reaction catalyzed by the mitochondrial enzymes GABA transaminase (GABA-T) and succinate semialdehyde dehydrogenase (SSADH). Succinate, being an intermediate component of the tricarboxylic acid (TCA) cycle, is subsequently converted to glutamine (Gln) by glutamine synthase, and Gln is then transported into neurons where it undergoes conversion to glutamate (Glu) [80]. It has been estimated that GABA metabolism accounts for ~8–10% of the total flow through the neuronal TCA cycle [80]. Most of this glutamate then undergoes conversion to glutamine (Gln) through the action of the strictly astrocyte-specific enzyme glutamine synthetase (GS) [81]. Gln is released by astrocytes and taken up by the closely apposed presynaptic neurons, where it can be re-converted to glutamate through the action of the predominantly neuronally localized enzyme phosphate-activated glutaminase (PAG) [82,83,84]; this process is regulated by the availability of phosphorylated species such as ATP and GTP [85,86], by TCA cycle intermediates [86,87], by cyclic nucleotides such as cAMP and cGMP [86], and by the products of the catalytic reaction (Glu and NH_4_) [86], allowing for negative control of this process at a number of different levels. Glutamate is thus readily available for GABA synthesis by GAD.

### 2.3. Mechanisms of GABA Transport and Synaptic Uptake

The GABAergic system plays an essential role in the fine temporal control of neuronal activity, at the level of individual neurons as well as larger neuronal populations. For this reason, the timing of receptor activation is important, and GABA levels in the extracellular compartment must be carefully regulated [88]. The clearance of GABA from the synaptic cleft, and its reuptake into neurons and astrocytes following neurotransmission, occurs through high-affinity GABA uptake systems [89,90]. GABA transport is mediated primarily by four GABA/Na^+^/Cl^−^ symporters—in humans these are GABA transporter 1 (GAT1), GABA transporter 2 (GAT2), GABA transporter 3 (GAT3) and the betaine-GABA transporter (BGT1). Within neurons, GABA transport into synaptic vesicles is mediated by the vesicular GABA transporter (vGAT) [91]. Functionally, the GABA transporters are responsible for the modulation of GABAergic inhibition by terminating the synaptic action of GABA and thus shaping the postsynaptic response to inhibitory presynaptic neurotransmitter release [92,93,94,95,96,97,98]. The differing ionic and pharmacological sensitivities of the different GABA transporters, and their differing affinities for GABA transport, make this a very heterogeneous uptake system which can control inhibitory synaptic signaling in a number of ways [99,100,101]. The GABA transporters are widely distributed throughout the mammalian central nervous system, with individual transporters having unique and sometimes overlapping regional distributions, mainly located on neuronal and glial cell membranes [102,103,104,105,106,107,108,109,110].

GAT1 is the most highly expressed GABA transporter in the mammalian cerebral cortex [111], and is generally considered to be the primary presynaptic neuronal GABA transporter. This transporter is also localized to astrocytic membranes at GABAergic synapses [104]. The extensive nature of GAT1 expression reflects its importance in regulating cortical excitability and information processing at synapses [104]. GAT2 is primarily found in various tissues outside of the CNS (in particular in the proximal tubules of the kidney, in the heart, and in liver hepatocytes), but has a limited distribution in some regions of the brain and in the retina [99,112,113]. GAT3 is primarily found in the nervous system, and is localized almost entirely to the processes of astrocytes within the cerebral cortex, indicating that this transporter is responsible for the uptake of GABA into astrocytes rather than neurons [114]. BGT1 is primarily a transporter for betaine, and has a lower affinity for GABA than the aforementioned transporters. The distribution of BGT1 in the mammalian brain is contentious, but some studies report that it is expressed by astrocytes at extrasynaptic sites, possibly suggesting a role in the regulation of tonic extracellular GABA levels.

### 2.4. GABA Receptors

GABA and its structural analogues bind to and activate GABA receptors (GABARs). The regulation of neural function by GABA occurs through its interaction with two classes of receptors—ionotropic GABA_A_/_C_ receptors and metabotropic GABA_B_ receptors.

GABA_A_ ionotropic receptors (GABA_A_Rs) are most commonly possess a pentameric structure, with five protein subunits arranged around a central Cl^−^ channel [115,116,117]. These receptors may be assembled from many possible subunit isoforms, each of which may belong to one of eight known subunit families—α, β, γ, δ, ρ, ε, θ and π [118,119]. Many of these subunit families comprise multiple subtypes (α_1–6_, β_1–4_, γ_1–4_, ρ_1–3_, ε, δ, θ and π), encoded by over 20 different genes [120,121]. There is a high degree of amino acid sequence identity within each subunit family (~70–80%), but much lower between them (~20%). Adding to this remarkable heterogeneity between and within subunit families, some of these subunits (such as γ_2_) may also exist as multiple splice variants [122,123]. Most GABA_A_Rs have a specific 2α:2β:1γ heteropentameric structure. In addition to this, each of these subunits may belong to any of the subtypes within their subunit family, and different variations of the 2α:2β:1γ template may occur in different brain regions [116,124,125,126]: ~60% of cerebral GABA_A_Rs are of the α_1_β_2_γ_2_ configuration, with the other two often-observed configurations being α_2_β_3_γ_2_ (~15–20%) and α_3_β_n_γ_2_ [127,128]. Other less common configurations are observed in brain regions such as the dentate gyrus (DG), hippocampus and cerebellum, and many other potential heteropentamers have been generated under laboratory conditions. Thus, there are many ways in which GABA_A_R subunits can interact—in fact, taken together, previous studies investigating the range of different possible GABA_A_R subtypes seem to point towards a remarkable promiscuity in the association of receptor subunits. Within receptor heteromers, different subunits are found at varying frequencies. Sieghart et al. have reviewed the methods commonly utilized to elucidate the subunit composition of the various possible receptor configurations [129]. It has been shown conclusively, in immunoprecipitation studies, that the α_1_ subunit is the most widely expressed, present in ~70–90% of GABA_A_Rs [129]. Another two subunits of the α-family, α_2_ and α_3_, are respectively present in approximately 35% and 14% of GABA_A_Rs [129]. Alternatively, α_5_ and α_6_ are less common, and found in only a small proportion of GABA_A_Rs, in particular in the hippocampus and cerebellum [129,130,131]. All three β-family subunits appear to occur quite commonly in subunit heteromers throughout the brain [126,129,131,132]. However, of the γ-family subunits, the γ_2_ subunit is by far the most commonly associated with other subunits in receptors (~50–60%) [129,133,134]. Multiple subunits from the same family may be present within the same receptor subtype.

Due to the ionic basis of the channel function of GABA_A_Rs, they are responsible for the inhibition and modulation of fast synaptic transmission. In general, GABAergic inhibition may be phasic or tonic. Phasic inhibition is produced by transient GABA release at the synapse, whereas tonic inhibition results from prolonged or continuous stimulation of extrasynaptic GABA_A_Rs by extrasynaptic GABA [135]. The extensive molecular heterogeneity of GABA_A_R subtypes, and the resulting array of potential receptor configurations, plays a central role in the modulation of inhibitory postsynaptic potentials (IPSPs). The specific subunit composition of postsynaptic GABA_A_Rs on the contacted domain of the neuron determines the nature of the response to inhibitory signaling, and the functional properties of GABA_A_Rs differ depending on their subunit compositions [136,137]. The orthosteric GABA binding site is located at the interface between the α and β subunits, and alterations in α and β subunit subtypes in the GABA_A_R may alter the affinity or sensitivity of the receptor to GABA and other orthosterically binding agonists and antagonists [138,139]. In addition, various modulators of the GABA_A_ receptor bind at allosteric sites—benzodiazepines (BZ), for instance, bind to the receptor at the γ_2_-α interface [129]. Thus, the absence of the γ_2_ subunit makes the receptor insensitive to this class of modulators, including drugs and endogenous ligands [140]. Further, γ subunits have also been shown to disrupt the Zn^2+^ interaction site on the GABA_A_R, and the absence of this subunit thus increases endogenous Zn^2+^ inhibition of GABA_A_Rs [141]. The γ_2_ subunit is also believed to play a role in GABA_A_R desensitization [142]. It has been shown that receptors containing the α_1_ subunit undergo rapid deactivation, whereas α_2_ subunit–containing receptors undergo slower deactivation [143]. Further, δ subunit–containing receptors are mostly extrasynaptic, and the δ subunit increases the sensitivity of the receptor to GABA, thus playing an important role in tonic inhibition [144]. Other endogenous and exogenous modulators of GABA_A_R function, including barbiturates, ethanol, steroids, picrotoxin, and melatonin, interact with the receptor at their various orthosteric or allosteric binding sites and function through diverse mechanisms of action [145]. Thus, the individual GABA_A_R subunits may each be involved in a range of functions, and they may interact with each other in complex ways. Interestingly, it has been shown that the GABA_A_R subunit composition may vary between different brain regions [129,146], and that subunit distributions may be altered in various neurological disorders [147,148,149]. Thus, it is clear that the pharmacological properties and sensitivities of the GABA_A_Rs are dependent on subunit composition. An understanding of GABAergic function in any specific brain region or cell type requires the knowledge of local GABA subunit distribution. It is thus essential to understand subunit alterations in health and disease in order to arrive at a comprehensive understanding of the impact of GABAergic dysfunction on brain function.

The functional GABA_B_ metabotropic receptor (GABA_B_R) incorporates two subunits—GABA_B1_ and GABA_B2_ (GB1 and GB2), both of which play distinct functional roles, and interact allosterically to regulate receptor function [150,151]. Like other GPCRs, the GABA_B_R subunits each consist of seven transmembrane α-helical domains, connected by intracellular and extracellular loops [152,153]. These proteins also feature an intracellular C-terminus and an extracellular N-terminal tail. The C-terminal region and the intracellular cytoplasmic loops are associated with a membrane-associated heterotrimeric G-protein, and this intracellular domain is also the site of interaction with a variety of other proteins, including adapter proteins and enzymes [154,155,156]. The GB1 subunit is responsible for the recognition and binding of ligands to the receptor via a large extracellular ligand-binding domain, but is not itself capable of forming a functional receptor [157,158]; in fact, the GB1 subunit features an endoplasmic reticulum (ER)-retention signal on its C-terminus, which prevents its trafficking to the cell membrane in vivo [159]. GB2 is required to ensure the correct trafficking of the receptor complex to the plasma membrane, through the masking of the GB1 ER-retention signal via an interaction between the C-terminal domains of both subunits [159]. In addition, signal transduction in GABA_B_Rs following the binding of agonists to the GB1 subunit is mediated by GB2—without this subunit, it is possible that the GABA_B_R is non-functional [150,160,161,162]. However, there is emerging evidence that the GB1 receptor subunit may have some functional capacity on its own—exerting effects on the ERK pathway, for instance [163]. The GABA_B_R may incorporate one of two isoforms of the GB1 subunit; these are termed GB1a and GB1b, and display differing distributions within the synapse [151,164,165]. GB1a-containing GABA_B_Rs are targeted to both axons and dendrites, as well as to glutamatergic terminals, whereas GABA_B_Rs incorporating GB1b are only trafficked to GABAergic dendritic terminals, and both receptor subtypes modulate differing functions at these sites [166,167,168]. These two GABA_B_R subtypes also have different expression profiles in various brain regions and at different stages of development [165]. Thus, GABA_B_Rs also display a small amount of structural heterogeneity which is of importance to the functional diversity of these receptors.

Our current understanding of GABA receptor alterations with respect to AD will be covered in detail in the next section.

## 3. GABA System Changes in Alzheimer’s Disease (AD)

### 3.1. Alterations in GABA Levels and Glutamic Acid Decarboxylase (GAD) Enzyme Activity

The role of GABAergic dysfunction in the pathogenesis of AD is a poorly understood and controversial matter. However, there is increasing evidence to suggest that neurotransmission at GABAergic synapses is significantly affected in AD in several different ways. Many studies have been concerned with the measurement of post-mortem GABA concentrations, GAD distribution and GAD activity in Alzheimer’s disease. However, the results of these studies, when taken together, are difficult to interpret due to their often-contradictory results, as well as the substantial limitations associated with post-mortem GABA and GAD measurement. In general, most post-mortem studies in tissue from patients with AD and other conditions with AD-like pathology indicate moderate-to-significant reductions in GABA concentrations in various cortical areas, including in the temporal [169,170,171,172,173,174,175,176], frontal [170,172,173,177,178], parietal [169,170,172,173] and occipital [173,175,177,178] cortices.

More specifically, within the frontal cortex, GABA depletion has been observed in the orbitofrontal region [178] and the premotor cortex [173]. However, several other post-mortem studies have indicated that GABA levels in one or more of these regions are not significantly altered [170,172,177,178,179], and GABA levels are reportedly unchanged within the insular and angular cortices [178]. One recent study found that GABA levels were substantially reduced within the AD temporal cortex, without looking into whether these changes are subregion-specific [176], but other studies generally report a decrease in GABA levels across multiple subregions of the temporal cortex [169,170,171,172,173,174,175].

Outside of the cortex, one study has reported reduced levels of post-mortem GABA in the cerebellum [175], while another reported no such changes [174]. Changes in post-mortem hippocampal GABA levels are similarly contentious, with some groups reporting decreases in the measured levels of the neurotransmitter [172,174,177] and some reporting a preservation of GABA levels [173,178,179,180]. One study also found decreased GABA levels in the parahippocampal cortex [173]. Within the AD thalamus, it has been observed that GABA levels are depressed in the dorsomedial nucleus [173], maintained in the anterior nucleus [173,174] and conserved [173,174] or reduced in the ventrolateral nucleus [178]. Another study has reported unchanged GABA levels in the thalamus in general [175]. Decreased GABA levels have also been observed by some in the cingulate cortex [173,178] and amygdala [172,178]. Other studies have reported that GABA levels are conserved in the putamen [173,174], caudate nucleus [173,174,175], substantia nigra [174,177,178], nucleus accumbens [173,174,178], amygdala [174], septal nuclei [174], hypothalamus [174,178], and cingulate cortex [170,174] in the AD brain. Only one study has actually reported a significant increase in GABA concentrations within the caudate nucleus [178], but GABA levels otherwise appear to be well-preserved in the structures of the basal ganglia in AD. As can be seen, many studies of GABA level alterations in the AD thalamus and subcortex are contradictory. However, it seems that there is a general trend in these studies towards a depression of GABA levels in many brain regions in AD. Reported changes in GABA levels in the aforementioned regions and others have been summarized in Table 1.

Suggesting the possibility of GABA upregulation in some regions, a recent study by Wu et al. demonstrated the anomalous accumulation of GABA in DG reactive astrocytes in post-mortem human AD tissue and in an AD mouse model (5xFAD) [181]. It was theorized that GABA is released through astrocytic GAT3 (GAT4 in mice), potentially resulting in raised extracellular GABA concentrations in this region [181]. As the authors point out, it is important to keep in mind that total GABA concentrations were measured in brain tissue homogenates containing both astrocytes and neurons, although it is likely from these results that increases in total GABA are driven by astrocytic synthesis/uptake and release. This may lead to increased ambient GABA levels, but might not influence the total GABA levels within the hippocampus, and it is important to note that this increase may be brain region- or subregion-specific. This could potentially be supported by the recent finding by Mitew et al. that in APP/PS1 transgenic mice, astrocytes may increase GABA synthesis in response to high regional Aβ load [182].

After considering the studies listed above, it becomes clear that there is little consensus in the literature regarding alterations in GABA levels in different regions of the AD brain. While some studies demonstrate significant and, in many cases, considerable reductions in GABA levels in various brain regions, these results are not always replicable. Some of these studies have been listed and reviewed in detail previously by Lanctôt et al. [183]. Many of these studies differ significantly in various aspects of study design, including sample size, the mean age of cases, gender, post-mortem delay, stage of the disease, comorbidity, cause of death, and use of CNS medications by patients prior to death. A common limitation in such studies is the availability of appropriate tissue; many groups lack access to tissue from defined subregions and from a large enough sample of patients to exclude those with confounding pre-conditions. This is important, as GABA levels may be influenced by a variety of different factors. Known prior use of CNS drugs (including BZs [171]), or a lack of records concerning prior drug use, may be a major confounding factor in many of these studies. Patient age and the stage of the disease are also important considerations—in a previously cited study by Rossor et al., it was demonstrated that hippocampal GABA levels were only decreased by about 24% in a cohort of 49 AD patients compared with controls, but an alteration of 41% was observed for AD patients below 79 years of age (*n* = 26) and only 9% for those above 79 years (*n* = 23) [172]. It thus becomes clear that in order to arrive at a conclusive understanding of alterations in GABA and GAD activity, a far greater degree of standardization is required in future studies, and the aforementioned confounding factors must be controlled for carefully where possible.

A major limitation with post-mortem studies of this nature is the effect of the antemortem agonal state—the period between the onset of the terminal phase of an illness and death due to the illness. It is well known that in this time, several important parameters may be significantly altered, including RNA and protein stability/degradation, tissue pH, enzyme activity and the levels of several biomolecular markers, and this can be especially relevant in the period immediately preceding death [184,185,186,187]. There is limited evidence to suggest that subcortical GABA levels are relatively unaffected by the agonal state [188], but that cortical GABA levels may be reduced [170]. The effect of the agonal state on post-mortem pharmacological and biochemical measures is dependent on the nature of the agonal state (disease severity, comorbidity, length of the agonal state, drug treatment, age, cause of death), and the precise circumstances and nature of the agonal state often vary considerably between patients and between experimental cohorts [189]. The agonal state may be particularly protracted in dementia, where death may commonly be attributed to bronchopneumonia [190]. Another concern relevant to these studies is the post-mortem rise in GABA levels in the mammalian brain, with levels increasing from 30 min to a few hours after death, and remaining stable for ~24–48 h after [191,192]. This makes the post-mortem interval (PMI) an important confounding factor in these studies, and the matching of cases by PMI is essential. Thus, potential antemortem and post-mortem changes in GABA levels make it difficult to study any dysregulation in GABA levels that might occur in AD. Several studies have attempted to address this concern by conducting antemortem studies of GABA in the cerebrospinal fluid (CSF). Most studies of this nature seem to indicate a preservation of GABA levels in the CSF of AD patients when compared with healthy controls [193,194,195,196,197,198,199,200]. However, some studies have demonstrated decreased CSF GABA concentrations in AD patients [201,202,203,204]. Aside from the confounding factors already mentioned, it is doubtful whether the results of these studies are neurologically relevant. GABAergic inhibition in the brain is dependent on synaptic and interstitial GABA, and tightly regulated by synaptic and extrasynaptic GABA transporters. GABA levels in the CSF are thus unlikely to be representative of physiologically important concentrations in the neuronal milieu and within the synaptic cleft.

Another measure of GABA system function is the activity of the GAD synthesizing enzyme. It was observed by Perry et al. that GAD activity was apparently reduced in post-mortem tissue homogenates (from all four cortical lobes) from patients with dementia (including AD and mixed-type dementias), but this was only observed at later stages of the disease, and was later shown by the same group to most likely have been the result of perimortem agonal state conditions (i.e., at or near the time of death) [205,206]. Indeed, GAD activity may be significantly affected by factors in the antemortem agonal state; among demented patients, this is often associated with decreased cerebral blood flow and respiratory conditions such as bronchopneumonia [206,207], conditions that have been associated with reduced GAD activity in the human and primate cerebral cortex [208,209]. In particular, GAD activity is affected by more acidic pH conditions in the agonal state [207]. This could well explain, at least in part, the sometimes-dramatic reductions in cortical GAD activity seen in some early studies of patients with dementia [210,211]. Indeed, this appears to be supported by a study by Reinikainen et al., in which cases were carefully selected based on a pre-mortem severity index (PMSI) to reduce the effect of the agonal state and of variable agonal state conditions between cases; in this study no significant changes were observed in GAD activity in any of multiple regions of the cerebral cortex, hippocampus, thalamus, pons and basal ganglia tested [212]. Thus, it is doubtful whether post-mortem measures will improve our understanding of GAD activity alterations in AD. Other studies have approached the problem of GAD dysfunction by examining GAD expression levels in the AD brain, as it has been demonstrated that GAD67 post-mortem mRNA levels are unaffected by antemortem conditions [213]. In an in situ hybridization study, Gao and Moore found no changes in the regional or cellular expression or distribution of cDNA for either of the two GAD isoforms within the human AD suprachiasmatic nucleus and the surrounding areas of the chiasmatic hypothalamus and retrochiasmatic area [214]. A recent study by Schwab et al. reported a severe reduction in GAD65 immunoreactivity in the human AD middle temporal gyrus (MTG), hippocampus and putamen, and confirmed with Western blot that GAD65 protein levels in the MTG were similarly reduced. However, they did not observe the same for GAD67 [215]. It should, however, be pointed out here that only a small proportion of GABAergic neurons in the basal ganglia were stained for GAD in this study, and it is thus possible that these results are only representative of one or more neuronal subpopulations that have been labeled with this antibody [215]. Another in situ hybridization study showed that GAD67 mRNA expression in the AD striatum was increased in the dorsal striatum and present in more neurons there, but no alteration was observed in the ventral striatum [216]. These changes were attributed not to an increase in the hybridization signal per cell, but an increase in the number of cells expressing GAD67 mRNA. However, the case number was small and it was not shown whether there was any correlation with protein expression [216]. An important point concerning these studies is that they did not consider GAD activity or expression with relation to local AD pathology. Mitew et al. demonstrated approximately doubled synaptosomal GAD activity in synaptosomal preparations from Aβ plaque–rich sites in the APP/PS1 transgenic mouse cortex, but no change in regions of the cerebellum that were plaque-free [182]. This could perhaps indicate localized GABAergic dysfunction in regions of the brain with more extensive AD pathology, and it will be important to confirm this in future studies.

Thus, it is fair to say that our understanding of AD-associated changes in GABA and GAD has been marred by limited comparability between studies, due to differing experimental conditions and case selection protocols, variable sample characteristics, differing (and often small) sample sizes, variable post-mortem intervals, and differing antemortem agonal state factors. Taken together, there appears to be a general trend towards the depression of GABAergic signaling and metabolism in many brain regions in patients with AD. Still, given the aforementioned concerns, future studies in this area will need to exercise great care in case selection and experimental design so as not to add to the large amount of inconclusive and uninterpretable data currently available.

### 3.2. GABAergic Neurons and Synaptic Dysfunction in AD

Based on a few early studies indicating a relative sparing of GABA levels and GAD activity in the AD brain [174,205,212,217], it has long been believed that GABAergic neurons are relatively spared during the pathogenesis of the disease. More recent studies have indicated neuronal sparing as well [218]. However, the assertion that GABAergic neurons are completely unaffected by the disease process has now been questioned in recent research. It has been demonstrated that there is considerable loss of GABAergic synaptosomal uptake in AD, indicating damage to GABAergic terminals independent of any actual GABAergic neuronal loss [217]. Thus, despite the apparent sparing of the GABAergic neuronal system, there is the potential for disruption in synaptic function at GABAergic terminals, which has been implicated as an important factor in the pathogenesis of AD and associated dysfunction in other neurotransmitter systems [47]. The binding of [^3^H]nipecotic acid, a radioligand based on an inhibitor selective for high-affinity GABA reuptake sites on synaptic terminals, is significantly reduced in the temporal cortex of the AD brain, but not in the frontal cortex or basal ganglia [219]. This could represent synaptosomal degeneration in the AD temporal cortex, or perhaps the downregulation of GAT1, and a decrease in GAT1 has indeed been previously observed in the mouse superior temporal cortex [218]. Bell et al. also demonstrated a significant reduction in GABAergic presynaptic bouton density in APP/PS1 (18-month-old) transgenic mice [220]. This appears to contradict the results of an earlier study by the same group, which observed a significant increase in GABAergic presynaptic bouton density in a TgCRND8 (4-month-old) mouse model of AD [221]. However, it is important to keep in mind that TgCRND8 mice display an early-onset form of the disease, exhibiting more severe memory deficits and Aβ deposition at an early age [222,223]. It is possible that early deficits in excitatory neurotransmission in TgCRND8 mice result in an upregulation of the presynaptic GAD65 marker used to label these boutons. Also important is the difference in age between the animals used in the two studies, which could imply that presynaptic bouton density is increased early in the course of disease pathology and decreases later in the disease. Ultimately, the differing characteristics of these mouse models make it difficult to compare these results in a meaningful manner. Contrary to these observations, Mitew et al. showed in a recent study that the density of presynaptic boutons expressing vGAT in the inferior temporal cortex is preserved in both early preclinical and late-stage forms of human AD, as well as in APP/PS1 (12-month-old) transgenic mice [182]. Bell et al. utilized GAD65 as a marker of GABAergic presynaptic boutons in their studies, while Mitew et al. used vGAT for this purpose. One has to consider the possibility that it was GAD65/vGAT protein expression changes that were measured in these studies rather than changes in bouton density, making it difficult to conclude from these studies whether and how presynaptic bouton density is affected. However, an interesting observation in the study by Mitew et al. was that vGAT-positive synaptic boutons in regions with high Aβ plaque density were larger in both preclinical and end-stage human AD when compared with controls. The authors theorize that this could indicate a larger pool of presynaptic GABA-containing vesicles, possibly representing a compensation mechanism for decreased excitatory signaling [182], but this may support the possibility of synaptic dysfunction in GABAergic neurons. It should be said here that the possibility of localized or selective interneuron loss cannot be completely discounted. In a recent study, it was shown that APP/PS1 mice exhibit significant hippocampal parvalbumin- and calretinin-positive interneuron loss in the CA1 and CA2 subfields, the DG and the hilus [224]. This was also observed in the post-mortem human DG, suggesting the possibility of population-specific GABAergic neuronal loss in the AD brain [224].

Bell et al. reported the relative resistance of GAD65-positive GABAergic neurites in APP/PS1 (18-month-old) and TgCRND8 (4-month-old) mice to dystrophy, even in regions of high Aβ load (in contrast to glutamatergic and cholinergic neurites, which were significantly dystrophic in these regions) [220]. Furthermore, it appears that the extent of GABAergic dystrophy in TgCRND8 mice is not influenced by plaque size, whereas larger plaques seem to cause greater dystrophy in glutamatergic and cholinergic neurons [221]. This led to the suggestion by this group that GABAergic terminals are more resistant to Aβ pathology than glutamatergic and cholinergic terminals, and likely become dysfunctional later in the course of the disease. However, in a study by Garcia-Marin et al., it was observed that there was a complete absence of GAT1 and vGAT-positive processes surrounding the soma of pyramidal cells in regions that were in contact with Aβ plaques, in both the AD human cortex (multiple regions) and in that of APP/PS1 (12-month-old) transgenic mice (multiple regions), while pyramidal cells which did not contact plaques exhibited perisomatic GAT1/vGAT labeling [225]. This could possibly result in hyperexcitability in pyramidal cells in the absence of GABAergic inhibition [225]. It could also be the case that the examination of the total change in presynaptic density masks more localized GABAergic terminal loss related to Aβ pathology. This could explain the observation by Mitew et al. that vGAT-positive boutons are preserved in the human AD cortex [182].

Some have theorized a specific role for GABAergic interneuron dysfunction in the early stages of AD. It has been found that a subpopulation of small calbindin-positive GABAergic interneurons in the hippocampus and temporal cortex upregulate nitric oxide synthase (NOS) expression in early AD, especially in areas of early tau pathology [226]. The authors point out that this could result in increased nitric oxide–induced apoptosis and neurodegeneration in pyramidal cells, and suggest that the NOS reactivity in these interneurons could serve as a marker of cortical dysfunction and neurofibrillary pathology in very early stages of AD [226]. Indeed, a correlation was observed between the numbers of these interneurons and the apoptotic marker caspase-3 [226]. In another recent study, it was found that a long variant of the Munc18-1 protein (M18L) is important for presynaptic GABA function, and that expression of this protein is reduced in the AD frontal cortex [227]. The extent of this loss seemed to be correlated with greater cognitive decline and more severe AD pathology, and a statistical regression model suggested that M18L loss was correlated with a higher likelihood of developing dementia. Based on this, and the fact that lowered M18L levels seem to increase cognitive sensitivity to AD pathology (and vice versa), the authors theorized that lowered levels of this GABAergic interneuron–specific protein may contribute to early cognitive decline. This could implicate biochemical changes at the GABAergic presynapse in the early pathogenesis of AD.

It is important to note at this stage that experimental results from transgenic AD mouse models, while informative, are very unlikely to accurately model the complex pathological, biochemical and behavioral changes inherent in the human condition. For instance, the APP/PS1 and TgCRND8 transgenic mouse models of AD do not display the tau pathology seen in human AD [222]. Considering the theorized contribution of tau pathology to the pathogenesis of AD, it is likely that these models do not accurately represent the synaptic pathology of the disease [228,229]. Indeed, these models display differences in behavior and disease progression, indicating differing functional consequences for the differing models of pathology [222]. In addition, there are significant differences in cortical circuitry, neuroarchitecture and neurochemistry between healthy humans and wild-type mice [230,231,232]. Thus, while such results are no doubt informative, one must be cautious in drawing too close a correlation between synaptic changes in animal models and the diseased human brain.

### 3.3. Alterations in GABA Receptor Distribution and Subunit Composition in AD

Several biochemical and molecular changes in the GABAergic system have also been reported in the AD brain, in particular in the structures, distributions and compositions of GABA_A_Rs in various brain regions (summarized in Table 2). It has been reported that GABA_B_R densities are reduced in the superior frontal gyrus of the AD cortex, despite the binding affinity remaining unchanged [233]. In this study, a modest but non-significant decrease in GABA_A_R sites was also noted [233]. The GB1 subunit in particular has been observed to be transiently upregulated with increasing tau pathology (i.e., later Braak stages) in the human AD hippocampus before being returned to relatively normal levels at later stages of the disease, perhaps indicating some kind of short-term compensatory change [234]. Of interest, studies have demonstrated potential changes in the protein and mRNA expression levels of specific GABA_A_R subunits in various regions of the AD brain. The α_1_ subunit, for example, has been found through immunohistochemistry to show decreased expression in the CA1, CA2 and prosubiculum fields of the human AD hippocampus [235]. Rissman et al. examined plasticity in the expression of hippocampal GABA_A_Rs at different stages of disease progression [236]. In this study, it was found that there were no significant changes in the protein expression levels of α_1_, β_1_ or β_2_ subunits in post-mortem tissue examined between Braak stages I to VI in any of the hippocampal regions studied [236]. However, it was observed that there was a modest decrease in α_5_ subunit expression in the CA1, CA2 and CA3 regions of the hippocampus when comparing between mild/moderate and severe AD cases [236]. In a subsequent study, the same group reported moderate the downregulation of α_1_ and α_5_ subunit mRNA in the post-mortem hippocampi of patients with probable AD [237]. However, an autoradiography study demonstrated that α_5_ subunit–containing GABA_A_Rs are relatively spared in the human AD hippocampus, with moderate decreases in density seen only in the CA1 region and the entorhinal and perirhinal cortices [238]. Mizukami et al. reported the preservation of β_2_/_3_ immunohistochemical labeling in all regions of the AD hippocampus at all stages of the disease, using an antibody recognizing both subunits, but this group also reported that hippocampal β_2_ mRNA was preserved and β_3_ mRNA reduced in late-stage AD [239,240]. Possible increases in γ_1/3_ protein levels in the hippocampus, using an antibody recognizing both proteins, have also been demonstrated in end-stage AD, with γ_2_ levels reportedly well-preserved [241]. Changes in GABA_A_R subunit expression have been observed in the human cerebral cortex as well. Luchetti et al. reported a decrease in GABA_A_R α_1_, α_2_, α_4_, β_2_ and δ subunits, the GABA_B_R R2 subunits and GAD1 mRNA levels in the mid-/late-stage AD prefrontal cortex, but no changes in GABA_A_R γ_2_, ε and θ subunits and the GABA_B_R R1 subunits [242]. Changes in the mRNA expression level and electrophysiological characteristics of a range of GABA_A_R subunits have been demonstrated in the post-mortem human AD temporal cortex (Braak stage IV to VI) compared with controls (Braak stage 0 to III). Significant decreases were observed in the mRNA expression of the α_1_, α_2_, α_5_, β_2_, β_3_, γ_2_, and δ subunits in the AD temporal cortex, and decreases in protein expression were observed in a Western blot study for the α_1_ and γ_2_ subunits; β_1_ and γ_1_ were unaffected at the mRNA or protein levels [243]. Independent of the observed downregulation in the absolute mRNA levels of these subunits, it was demonstrated that a higher proportion of GABA_A_Rs in the AD temporal cortex incorporated the α_2_, β_1_ and γ_1_ subunits compared with GABA_A_Rs in control temporal cortices, and a lower proportion of the GABA_A_Rs in the AD temporal cortex incorporated the α_1_ and γ_2_ subunits [243]. Considering the fact that some of the control cases in this study had stage II and III Braak pathology, it is possible the changes observed would have been larger had controls without neurofibrillary tangle (NFT) pathology been utilized.

Thus, it is clear that even within the human hippocampus and cerebral cortex, studies of receptor subunit mRNA and protein expression levels are difficult to interpret, and often contradictory. It is worth noting that modest changes in subunit mRNA expression, as noted by some studies, may not translate to significant changes in protein expression. This could well explain some of the disagreement between mRNA and protein expression studies, as well as the variability in the resulting data when expression changes are modest or minor. This has been proposed by Rissman et al. as an explanation for the fact that α_1_ and α_5_ downregulation at the mRNA level seem to be far greater than the observed corresponding change in protein [236,237]. Similarly, Mizukami et al. have noted a decrease in β_3_ mRNA levels in one study, but unaltered β_3_ protein expression in another [239,240]. This could also partially explain the relative sparing of the hippocampal α_5_ subunit in some studies and the modest mRNA downregulation in others [237,238]. Another interesting theory, proposed by Armstrong et al., is that the unchanged receptor subunit expression levels between healthy and advanced AD cases may represent an upregulation of the receptors, as neuronal loss would be expected in terminal stages of the condition [245]. Similarly, modest reductions in receptor mRNA or protein levels in late-stage AD may simply represent neuronal loss. Such considerations are very important when attempting to interpret this data, and it is thus important that immunohistochemical studies control for cell death, particularly in later stages of AD pathology; it is unclear from the results of many studies whether subunit changes represent changing neuronal density, changes in receptor subunit compositions or compensatory changes in GABA_A_ receptor and/or subunit density. For instance, Mizukami et al. observed that reductions in β_3_ mRNA levels occurred in both regions with little and significant neuronal loss, making it possible that some subunit changes occur before cell dysfunction and are predictive of vulnerability, whereas the preservation of β_2_ mRNA even in severely affected regions might indicate that β_2_-containing GABA_A_Rs are upregulated in surviving neurons or that early upregulation has a neuroprotective effect in neuronal subpopulations. Thus far, there have only been a few studies attempting to elucidate patterns of GABA receptor expression change in the human AD cortex and hippocampus, and further studies with good case control strategies will need to be conducted to provide more concrete answers here.

In addition to the direct measurement of GABA receptor density using immunohistochemical, autoradiographic and mRNA-based methods, radioligand binding studies have been conducted in which radiolabeled ligands for the allosteric BZ binding site on the GABA_A_R were used to estimate changes in BZ affinity in AD post-mortem tissue. A decrease in tissue affinity for BZs could indicate a downregulation of the α and/or γ subunits that constitute the BZ-binding interface. A number of these studies have been comprehensively surveyed by Lanctôt et al. [183]. The most commonly utilized radioligand in such studies has been [^3^H]flunitrazepam, and these studies have shown significant decreases in BZ binding affinity in the posterior middle temporal gyrus (layers I–V) [246], the frontal cortex [247], the temporal cortex [247,248,249], and the hippocampus [247]. Some of these studies have also reported the preservation of BZ affinity in the primary visual cortex and visual association cortex [246], the orbitofrontal cortex [246], layer II of the entorhinal area of the parahippocampus [250], and the stratum pyramidale-radiatum of the hippocampus [250]. While a study by Greenamyre et al. demonstrated a general preservation of BZ binding affinity within the parahippocampus [251], Jansen et al. demonstrated a decrease in affinity in layers III and IV of the entorhinal area of the parahippocampus and no changes in affinity within layer II [250]. The decrease in affinity observed by Shimohama et al. was not reported by Greenamyre et al. [251] and Jansen et al. [250], however Greenamyre and colleagues did observe a decreased B_max_, potentially indicating a loss of receptor density in this region, and Jansen and colleagues considered only the stratum pyramidale-radiatum. In the subcortical region, no changes in BZ binding affinity were observed in any of the studies that examined this [247,252]. It should be noted that none of the three studies of BZ affinity in the AD cerebral cortex examined regional differences within the temporal cortex, and unpublished observations from our lab have indicated regional differences in GABA_A_R subunit distribution in the temporal cortex, and region-specific alterations in AD.

It is important to note that there exist two different forms of the human BZ receptor. Apart from their function as allosteric modulators of the GABA_A_R, so-called peripheral benzodiazepine receptors (pBZR) are also present in mitochondrial membranes in all tissue types, including neurons and glial cells [253]. Thus, the affinity of BZ drugs such as flunitrazepam at the mitochondrial membrane is dependent on three components of the pBZR complex—the mitochondrial voltage-dependent anion channel (VDAC) [254,255], the adenine-nucleotide carrier (ADC) [254,255] and the mitochondrial translocator protein (TSPO) [256]; it is known that the number (B_max_) of these binding sites may be altered in conditions such as hypertension [257], and in response to kainate-induced injury [258], increases in VDAC1 mRNA expression have been observed in some brain regions in post-mortem human patients [259,260] and in an APP transgenic mouse model of AD [260], and astrocytic and vascular TSPO levels are disturbed in the AD brain [261]. Diorio et al. also demonstrated that pBZR binding sites (B_max_) are increased to a highly significant extent in the post-mortem AD temporal cortex, and also observed a similar increase in the frontal cortex that approached significance. They did not observe any changes in affinity in either region as measured by [^3^H]flunitrazepam binding [262]. Such changes in mitochondrial pBZRs in AD are perhaps not surprising, given the extent of mitochondrial dysfunction observed in this disorder. Appropriate plasma membrane extraction is essential in studies of BZ binding properties in AD, to avoid the contribution of mitochondrial pBZR alterations as a confounding factor.

The aforementioned binding studies are complemented by four neuroimaging studies (two positron emission tomography (PET) and two single-photon emission computed tomography (SPECT) studies) that have also attempted to investigate changes in GABA receptor affinity in the AD brain in live patients. These studies represent our only readout of GABA receptor changes in AD prior to death. The PET studies, utilizing ^11^C-flumazenil (a BZR inverse agonist) as a radioligand, measured binding as the difference between uptake in the brain and loss in washout fractions. Both studies seemed to indicate that BZR binding sites are well-preserved in AD [263,264]. Two SPECT studies have utilized ^123^I-iomazenil (a BZR partial inverse agonist) to measure BZR binding in the same manner. Wyper et al. noted that iomazenil binding was preserved in the frontal and temporal cortices and basal ganglia, but observed a small decrease in binding in the parietal cortex [265]. Fukuchi et al. observed the same in the temporal and occipital cortices, but noted a moderate decrease in iomazenil binding in the frontal and parietal cortices [266]. These studies seem to indicate that BZR density is not severely affected at the level of broader cortical regions, but a limitation of these studies is the inability to measure changes in more localized areas—the previously described post-mortem radioligand binding studies seem to indicate that sub-regional distributions may be altered. Nonetheless, these studies are still valuable as they avoid potential confounding factors introduced in the agonal state (e.g., the upregulation or downregulation of receptor subunits in response to agonal stresses in the brain). It is important to note, however, that these studies were often conducted with small sample groups, without controlling appropriately for factors such as age, gender, and concurrent medications or comorbidities [183].

Thus, although there have been conflicting reports on the specific changes in GABA_A_R subunit composition in AD brains, there is evidence to suggest that such alterations do occur. This would likely influence the pharmacological properties and sensitivities of the GABA_A_R, and thus the inhibitory neurotransmission in the AD brain. It is also possible that such changes occur in other brain regions that have not yet been studied. Previous studies have been inconsistent due to large variations in the quality of cases, different experimental techniques and conditions utilized. In addition, differences in agonal state changes between cases must be considered when comparing diseased and control cohorts, as well as uniformity of PMIs between cases. PMI may have a significant effect on the RNA expression of a range of different genes [267]. More uniform testing procedures and appropriate case-matching will thus be of significant benefit in this area.

### 3.4. GABA Transporter Alterations

Nägga et al. conducted a study in which they measured the displacement of the selective GAT1 radiolabel [^3^H]tiagabine by GABA in the human frontal and temporal cortices and the caudate nucleus; it was reported in this study that the total number of presynaptic GAT1 transporters and affinity for GABA were both unaffected in the AD tissue [244]. Another study presented immunohistochemical evidence in human AD brains and in an AD mouse model (5xFAD) for a significant increase in GAT3 in dentate gyrus reactive astrocytes [181]. An early study also reported the downregulation of high-affinity GABA reuptake sites in temporal cortex synapses but not in the frontal cortex or basal ganglia, measured by the binding of a GABA reuptake inhibitor, [^3^H]nipecotic acid [219]. This could indicate that GAT1 transporters may possibly be downregulated in this region, but could alternatively be a consequence of neuronal or synaptosomal loss. It should be noted, however, that nipecotic acid has been discovered to potentially interact with GABA_A_Rs [268], which may call into question the reliability of [^3^H]nipecotic acid binding as a measure of GABA transporter alteration.

There is currently only a single study in the literature examining concurrent changes in the expression of the three most common GABA transporters in post-mortem human AD tissue. Fuhrer et al. demonstrated that GABA transporter expression was differentially altered in various brain regions, and that differential expression could be observed in a layer-specific manner within the same brain region [218]. Utilizing fluorescence immunocytochemistry, it was demonstrated that BGT-1 density was significantly increased in the stratum radiatum of the CA2 and CA3 regions of the AD hippocampus and in all layers of the dentate gyrus, as well as in the superior temporal gyrus; no significant changes were observed in the CA1 hippocampal region, the subiculum or the entorhinal cortex [218]. It was also observed that GAT1 density was significantly reduced in the entorhinal cortex and superior temporal gyrus, with no significant changes observed in the CA1, CA2 or CA3 hippocampal regions, the dentate gyrus or the subiculum [218]. GAT3 staining was shown to be significantly decreased in the stratum pyramidale of the CA1 and CA3 regions of the hippocampus, the subiculum and the entorhinal cortex, with large but non-significant decreases in the CA2 hippocampus, dentate gyrus and superior temporal gyrus [218]. These changes did not appear in most cases to be strongly correlated with neuronal death, tau load or β-amyloid load, which may suggest a mechanism independent of these common measures of AD progression. Fuhrer et al. also examined GABA transporter expression in astrocytes in the aforementioned regions, and reported increased BGT-1 expression and a decrease in GAT3 density on astrocyte dendrites, processes and throughout the neuropil in the stratum pyramidale of the CA1 and CA3 hippocampal regions, the subiculum and entorhinal cortex; GAT1 exhibited a decreased expression profile in astrocytes within the superior temporal gyrus and entorhinal cortex [218]. The results of this study complement those of Nägga et al., as decreased GAT1 density was observed in hippocampal and cortical regions that were not examined in their study. The results of Fuhrer et al. regarding GAT1 downregulation in superior temporal cortex astrocytes are also in agreement with the previous study showing decreased synaptic GABA uptake sites in the temporal cortex [219]. However, the decreased GAT3 expression observed in the dentate gyrus by Fuhrer et al. is in opposition to the large increase in expression observed by Wu et al. [181]. Reported changes in GABA transporter expression levels in the AD brain are summarized in Table 2.

Considering the important role of GABA transporters in synaptic GABA clearance and the maintenance of extrasynaptic GABA levels, these reported changes in GABA transporter expression in AD could well contribute to the altered level of the neurotransmitter measured in human post-mortem and antemortem studies in various brain regions, potentially resulting in altered neuronal excitability patterns. Altered GABA transporter expression may result in the dysfunctional regulation of cellular osmolarity, in particular BGT1, which is also involved in the transport of osmotic regulators such as taurine and betaine [269]. One striking finding by Fuhrer et al. was the apparent potential for co-regulation in the expression of these transporters in some brain regions. It was found that decreased GAT1 expression in the superior temporal gyrus and decreased GAT3 expression in the CA3 hippocampus were accompanied by an increase in BGT1 density in these regions, and the authors of this study theorize that this could represent a compensatory mechanism for the maintenance of GAT activity in the AD brain [218]. A previous study demonstrated a similar pattern of GABA transporter expression changes in the healthy rat hippocampus following neuronal injury by kainate injection—they suggest that this may be a mechanism by which astrocytes respond to hyperosmotic stress, but which may result in excessive water intake as a result of prolonged upregulation [270]. This is an interesting hypothesis, considering the fact that BGT1 expression levels are generally lower than those of GAT1 and GAT3 in the mouse and human brain [271], that BGT1 appears to be more commonly expressed in the leptomeninges than in brain tissue [272,273], and that BGT1 has the lowest affinity for GABA among the three transporters [99,274]. It has thus been suggested that this might diminish the importance of this transporter in synaptic GABA clearance. The widespread localization of BGT1 has been reported at non-GABAergic sites, and it has been demonstrated by the co-application of a GAT1/BGT1 inhibitor with an extrasynaptic GABA_A_R-targeting anticonvulsant drug (gaboxadol) resulting in a decrease in the efficacy of this drug [275], suggesting a role for BGT1 in the regulation of tonic GABA stimulation at extrasynaptic sites. Thus, given the differing functional characteristics of BGT1 compared with GAT1 and GAT3, it is perhaps unclear whether the upregulation of BGT1 in certain brain regions in AD represents a compensatory response to GAT1/3 downregulation or osmotic stress. It should be noted here that Kempson et al. have pointed out that BGT1 control of osmolarity is suspect, due to the low concentration of betaine in the brain, the maintenance of steady betaine levels with osmotic stress, and the presence of other more powerful osmolyte transporters such as SMIT in astrocytes [276]. Keeping in mind the current uncertainty over the function of brain BGT1, further studies will be required to clarify the effect of changing GABA transporter expression patterns and the relevance of any potential compensatory mechanisms in the maintenance of synaptic and extrasynaptic GABA levels and cellular osmoregulation in AD.

### 3.5. Tau Pathology and GABA Signaling

Tau is a common microtubule-stabilizing protein in the mammalian brain, and plays a key role in regulating the stability of axonal microtubule structures [277]. These proteins are often found to be overexpressed and hyperphosphorylated in AD and related dementias, forming so-called neurofibrillary tangles (NFTs) within neurons. This can result in a loss of microtubule stability and neuronal death. Proponents of the tau hypothesis of AD pathogenesis hold that this abnormal aggregation and hyperphosphorylation is responsible for much of the neuronal death and dysfunction observed in AD, and much evidence points to tau as a central or important player in the pathogenesis of AD and related diseases [278,279,280]. A relationship between NFT formation and GABAergic dysfunction in AD has been suggested in several studies. NFT proliferation has been associated with changing the GABA_A_R subunit distribution within the human AD hippocampus. It has been reported in immunohistochemical studies that γ_1/3_ subunit density is reduced and α_1_ labeling is increased in Braak stages V to VI, while mean γ_2_ and β_2/3_ labeling appear to be preserved even at this advanced stage of NFT pathology [235,241]. Hippocampal β_2_ mRNA levels are reportedly stable in Braak stages V to VI, while β_3_ mRNA levels are reduced in Braak stages V to VI [240]. However, it is unclear whether such changes are due directly to tau pathology or some other aspect of disease progression. Indeed, it was reported that γ subunit–expressing hippocampal interneurons do not contain NFTs, which could potentially indicate that observed changes in γ_1_/_3_ density in this study are independent of tau pathology [241]. Alternatively, it could mean that these receptors are protective against NFT formation, or that neurons containing these receptors are more sensitive to NFTs and die early in the disease, or even that tau pathology in other nearby cells causes the downregulation of γ receptors in these interneurons through some indirect means such as signaling alterations at synapses. In considering the results of studies that have correlated the Braak stage with changes in the expression of specific GABA system components, it is not possible to determine whether worsening NFT pathology is responsible for these changes or some other progressive aspect of disease pathogenesis.

Some evidence for tau-modulated disturbance in the GABA signaling system has been derived from tau pathology–expressing animal models. It has been found in a triple transgenic mouse model (TauPS2APP), overexpressing *tau* genes, that GABAergic septo-hippocampal projection neurons underwent increased degeneration, along with the cells that these neurons contacted [281]. This degeneration in target cells was present in all areas that were examined, but interneurons of different classes and in different sub-regions appeared to be differentially susceptible [281]. It is important to note here that the relative contributions of the three gene mutations involved cannot be determined, and they likely interact in a complex manner. However, another study utilizing JNPL3 (BL6) *tau*-mutant mice demonstrated again the degeneration of GABAergic interneurons in aged mice [282]. In both studies, it was found that the mice exhibited enhanced long-term potentiation (LTP) in hippocampal slices, which in JNPL3 (BL6) mice could be rescued with the use of the GABA_A_R agonist zolpidem [281,282]. Aged JNPL3 (BL6) mice also displayed memory deficits [282], and both models displayed sensorimotor disturbances [281,282]. Another *tau*-mutant mouse model, P301L, expressing the long human tau40 isoform displayed disturbances in glutamate and GABA metabolism, with an increase in GABA turnover in cortical astrocytes and glutamate turnover in glutamatergic and GABAergic neurons [283].

Aside from the aforementioned animal studies, Nykänen et al. demonstrated that tau phosphorylation may be modulated directly by the activation of the GABA_A_R [284]. It was found that the treatment of GABA_A_R-expressing mouse Neuro-2A cells with BZs or barbiturates appeared to promote the interaction of tau with Pin1—a key regulator and facilitator of PP2A-mediated tau dephosphorylation [284]. Furthermore, treatment with the GABA_A_R agonist muscimol generated a similar increase in the tau-Pin1 association, which could be blocked with the administration of the GABA_A_R antagonist picrotoxin [284]. This group went on to show in mature DIV 21 rat cortical neurons that GABA_A_R-mediated signaling actually promotes phosphorylation at the Ser199/Ser202/Thr205 site in the tau protein through the action of the Cdk5 kinase [284]. However, in these cells, treatment with GABA_A_R modulators elicited a decrease in the tau-PP2A association [284]. Interestingly, the authors point out that phosphorylation of the GABA_A_R β_3_ subunit is important in the desensitization of GABA_A_Rs containing this subunit, and that this subunit is dephosphorylated by PP2A, potentially recruiting PP2A away from sites of tau phosphorylation. Indeed, some previous studies seem to have found a link between GABAergic anesthetic use and tau phosphorylation. For instance, sevoflurane, an anesthetic thought to act as both a positive allosteric modulator and antagonist at allosteric and orthosteric sites on the GABA_A_R [285], has been found to increase tau phosphorylation in C57B16/J mice, which was reversible with chronic administration and persistent with clear memory impairment after repeated administration [286]. Other anesthetics with confirmed or suspected GABA_A_R-targeted activity have also been associated with increased tau hyperphosphorylation, including propofol [287] and pentobarbital [288,289].

Taken together, the results of the aforementioned studies seem to indicate a clear role for GABA_A_R signaling in tau hyperphosphorylation in AD and related disorders, albeit one that needs to be investigated further.

## 4. Disruption of the Excitatory/Inhibitory (E/I) Balance in AD

Brain homeostasis and learning and memory formation depend on the maintenance of a dynamic balance between the GABAergic and glutamatergic systems, respectively the main inhibitory and excitatory neurotransmitter systems in the brain. In addition to atrophic changes in brain regions such as the hippocampus and temporal cortex [290], early aberrant excitatory neurotransmission is a well-attested phenomenon in both AD animal models [291,292] and AD patients [293,294]. However, as presented earlier in this review, GABAergic remodeling may be a previously overlooked feature of the disease, and may potentially contribute to pathogenesis.

Given that an optimal balance between excitatory and inhibitory network activity is necessary for learning and memory formation in cortical regions (the default network) and the hippocampus [295], and with our current understanding of the importance of GABAergic signaling in the synchronization of cortical network activity, it is possible that glutamatergic signaling changes are accompanied by GABAergic alterations in the AD brain, with both contributing to the E/I imbalance that underlies cognitive impairment in the disease. It has been shown that, in addition to the modulatory effect of GABA on glutamate release [296], signaling through glutamate and NMDA receptors can also exert a reciprocal effect on GABA release [297]. With the significant remodeling apparent in the glutamatergic system in AD, it is likely that this has a downstream effect on the GABAergic system. Reciprocal GABAergic responses to glutamate dysfunction could potentially account for the changes in GABAergic signaling and network activity that have been observed in AD and possibly contribute to the previously described molecular remodeling of the GABAergic system. Changing GABA levels could in turn have a significant impact on glutamate-induced excitotoxic cell death. Erdő et al. showed that 1–5 μM concentrations of GABA significantly accelerated excitotoxic cell death in cultures of rat cortical neurons [298], demonstrating the sensitivity of glutamatergic cells to ambient GABA levels. However, the mechanisms underlying these changes are not well understood.

As per the cholinergic hypothesis of AD, the dysfunction of the cholinergic system is also associated with early AD pathogenesis and the development of cognitive deficits [299,300]. Muscarinic receptors, specifically the M1 subtype, have been broadly related to AD. The M1 receptor is widely distributed in the brain and is expressed post-synaptically in the cortex and hippocampus [301,302]. Thus, early disruptions in GABAergic signaling, in association with impairments in glutamatergic and cholinergic excitatory signaling, could underlie the E/I imbalance in the AD brain, leading to subsequent network disruption [147,183].

Disruptions or alterations in GABA levels, GABA currents, GABA_A_Rs and GABA transporters, broadly covered earlier in this review, may have a great impact on hippocampal and cortical function, and thus in the progression of the disease. In addition to the previously described studies of total GABA level alterations in various post-mortem human brain regions, there is evidence from animal studies of altered extracellular GABA levels in AD. One study by Li et al. found that GABA release in hippocampal slices from mice expressing apoE4 (the main genetic risk factor for AD) was found to be diminished [303]. In addition, extrasynaptic GABA levels, dependent on synaptic spillover and the activity of GABA transporters, have been demonstrated to be altered in a 5xFAD AD mouse model [181]. Extracellular GABA levels have also been found to stimulate enhanced synthesis of APP, disruptions in neuronal membrane function and the promotion of neurodegeneration in the basal forebrain [298,304]. The pathological processing of APP and the aberrant deposition of Aβ is a well-known phenomenon in AD [1]. In past decades, much effort has been put into understanding Aβ-associated glutamate excitotoxicity involving the dysfunction of calcium-permeable glutamate receptors in the neuron [305,306]. Aβ has been implicated in the inhibition of glutamate uptake in the synaptic cleft [307], overstimulation of NMDA receptors, the subsequent disruption of calcium-dependent intracellular pathways [308] and cell death [309]. Neuronal cell death is preceded by mitochondrial fragmentation and impairments in energy metabolism, membrane lipid peroxidation, increased formation of reactive free radical species and oxidative stress [309,310,311].

Studies suggest that Aβ pathology has a direct functional effect on GABA_A_R-mediated currents, but such results have often been contradictory, likely as a result of vastly different study conditions and the different animal models and brain regions assessed. In combination with the effects of Aβ on glutamate-mediated excitatory currents [312], Aβ-associated alterations in GABA inhibitory neurotransmission could represent a key mechanism whereby network activity is impaired. It was found in one study that acute pre-treatment of rat hippocampal CA1 neurons with Aβ_25–35_ and Aβ_31–35_ actually enhanced GABA-induced electrical activity while suppressing glutamate-induced NMDA currents [313]. However, another study that measured GABA currents in AD human temporal cortex GABA_A_Rs that had been transplanted into *Xenopus laevis* oocytes demonstrated a ~70% suppression of GABA_A_R-mediated currents when compared with controls, similar to the AD-associated downregulation of glutamate currents [243]. It is interesting to note that hippocampal long-term potentiation (LTP)—a phenomenon associated with learning and memory—is demonstrably reduced or blocked by soluble Aβ_1–40_ oligomers that appear to exert their effect through both glutamatergic and GABAergic disruption [314]. In a recent study, acute Aβ_1–42_ administration resulted in the reduced generation of inhibitory post-synaptic currents (IPSCs) and a decreased post-synaptic response to acute GABA application [315]. The finding that this could be prevented with an inhibitor of GABA_A_R endocytosis led the author to conclude that Aβ may cause postsynaptic GABA_A_R downregulation [315]. However, given the fact that previous studies report preserved GABA_A_R density in post-mortem human AD cortical tissue, it is questionable whether this mechanism is relevant to alterations in GABA-induced electrical activity in human AD. It has also been found that Aβ_25–35_ treatment depresses GABA_B_R-mediated inhibitory post-synaptic potentials (IPSPs) in rat CA3 neurons, most likely through its effect on GABA_B_R-modulated inward rectifier potassium (GirK) channels [316] which play an important role in the regulation of neuronal excitability [317]. It has been shown recently that AD is associated with a ~10-fold upregulation within the neocortex of a non-coding RNA termed 17A, located within the GPR51 gene (coding for GABA_B_R subunit GB2) [318]. The 17A expression was shown in SHSY5Y neuroblastoma cells to induce the expression of the GB2b splice variant and decrease the expression of GB2a, ablating GABA_B_R-mediated signaling in these cells [318]. Thus, GABA_B_R signaling may be severely impaired in the human AD cortex. There have not been any investigations into whether 17A is expressed in wild-type or AD rodents, but this could represent another mechanism by which GABA_B_R-mediated IPSPs are impaired.

During neuronal communication and memory processing, GABAergic activity plays an important role in neuronal synchronization during theta and gamma activity in certain brain regions [319,320]. Specifically, gamma conductance disruption has been proposed as a potential mechanism of network hyperactivity, thus contributing to the (E/I) imbalance in AD [321,322,323]. A few groups have studied such network alterations in mouse models of AD, and the role of pathological Aβ. It was found that the brains of APP transgenic mice exhibited network hypersynchrony, especially during periods of reduced gamma oscillations [324]. Similar spontaneous epileptiform activity is often observed in early-onset heritable forms of AD, and less frequently in later-onset sporadic cases [325]. The authors propose that this network dysfunction could be caused by deficits in parvalbumin (PV)-positive interneurons, as the synaptic firing of these neurons underlies the generation of gamma oscillations [324]. Indeed, it was found that both hAPP mice, with high Aβ load, and human AD patients expressed less Nav1.1 compared with controls [324]. Nav1.1 is a voltage-gated sodium channel found primarily on PV-positive interneurons which contributes to the synaptic activity of these cells. The same group found that the restoration of Nav1.1 levels improved memory deficits in hAPP mice [324], leading the authors to suggest that behavioral or pharmacological therapies that reduce network hyperactivity or increase gamma activity could have potential in the treatment of Aβ-associated cognitive deficits in patients with AD [324]. hAPP mice exhibiting this spontaneous cortical and hippocampal epileptiform activity also show increased sprouting of GABAergic terminals, greater synaptic inhibition, and abnormalities in short- and long-term synaptic plasticity within the hippocampal dentate gyrus region; this network remodeling to potentiate GABAergic function is likely a response to Aβ-induced hyperexcitation [291]. It is possible that GABA release by neurons under network hyperactivity conditions might be a compensatory mechanism to overcome the glutamatergic overactivation induced by Aβ [326]. Notably, reduced GABA-induced Cl^−^ currents have been recorded in response to the application of Aβ_1–40_ and Aβ_25–35_ in *Aplysia kurodai* (a species of sea slug) [327]. This suggests that Aβ could have direct effects on GABA_A_R-mediated currents, and that GABAergic dysfunction could encompass both compensatory elements and direct pathological responses to Aβ. Indeed, chronic exposure to Aβ increases GABA activity, and as a compensatory mechanism, several GABA_A_R subunits seem to be upregulated or downregulated [313]. Conversely, acute Aβ effects might be related to an inhibitory deficit; specifically, a decrease in the GABA-immunoreactive neuron number has been observed after three days of Aβ incubation in rat basal forebrain cell cultures [328], indicating that GABAergic neurons might respond differently to acute or chronic Aβ exposure. Thus, despite some convincing evidence suggesting GABAergic vulnerability to Aβ in the early stages of AD [312,313,315,327,328], the relationship between GABA and the amyloid cascade hypothesis remains unclear.

Given the large body of compelling evidence related to changes in the two main neurotransmission systems in the AD brain, it is likely that these alterations underlie aberrant synaptic function and E/I imbalance, leading to deficits in learning and memory and synaptic vulnerability in AD.

Many neuroprotective compounds have been previously described with the ability to ameliorate glutamate-dependent excitotoxicity through the binding of GABA_A_Rs, possibly modulating and restoring E/I network balance [329,330,331]. Intriguingly, GABA itself, in combination with other compounds that bind to GABA_A_Rs, might prevent Aβ-induced neurotoxicity [329,330,332,333], and GABA_A_ agonists have demonstrated neuroprotective and anti-amyloidogenic effects in aged mice and cultured rat cortical neurons [331,334,335]. Various GABAergic drugs have been studied in relation to Alzheimer’s disease, and a small number of these have reached clinical trials [336]. Drugs targeting extrasynaptic GABA_A_Rs are currently being explored, with some being tested in pre-clinical and clinical studies for their potential to improve cognition [336,337,338]; these might offer a promising route in the symptomatic treatment of the disease. The possibility of targeting other GABA-mediated pathways is also being explored, with the GABA_A_R-targeting drug etazolate having been tested in Phase II clinical trials for its neuroprotective and anti-amyloidogenic effects in AD patients [336,339,340,341,342]. Attention is also increasingly being paid to the relationship between BZ use and the risk of developing AD [343,344].

Taken together, these point to the GABAergic system as a key regulatory factor, the therapeutic targeting of which might improve E/I balance in the AD brain, hence ameliorating the neurotoxic effect of Aβ and potentially restoring cognition in early or later stages of AD. The investigation of substances that act at GABAergic targets, and which protect directly or indirectly against neurotoxic Aβ by themselves and/or in combination with other drugs, is one promising direction for therapeutic research into AD. To better assess the suitability of the GABAergic system as a drug target in this disease, GABAergic dysfunction and its contribution to AD pathogenesis must be further elucidated.

## 5. Conclusions

In the past few decades, many studies have implicated the disruption of cholinergic and glutamatergic neurotransmission in AD. As described in this review, increasing attention is also being paid to the role of GABAergic dysfunction in this disease. Despite some controversy in the field, there is much evidence to suggest that GABAergic remodeling is a feature of AD, being potentially initiated at early stages of disease pathogenesis. There is evidence that alterations in various components of the GABAergic system, including GABA levels, GAD activity, GABA currents, and the distribution and subunit composition of GABARs and GABA transporters, might not be occurring simply as a compensatory mechanism in response to glutamate excitotoxicity. Indeed, some alterations might also be caused by the direct effect of Aβ. Thus, the Aβ-induced disruption of GABAergic inhibitory neurotransmission could represent a key mechanism whereby network activity is impaired in AD. In this manner, GABAergic remodeling may be involved in E/I balance disruptions that lead to early cognitive deterioration in the AD brain. A novel idea emerging from this body of research is the suggestion that the GABAergic system is an important factor in both the early and later stages of disease progression, and is not simply altered as a secondary pathological response. It is thus important to consider both direct and compensatory alterations in GABAergic activity in AD. Due to the limitations of previous studies discussed in this review, and the inconsistency of previous results, the consequences of these alterations on neural network activity and behavior/cognition are not yet well understood. It is also important to take into account the huge translational gap between animal and in vitro models of AD and human clinical trials, and to consider the possibility that currently available AD models fail to capture key characteristics of the human disease.

Therefore, there is an urgent need to pursue further research in this area, to enhance our understanding of AD-associated alterations in the GABAergic system. Evidence for AD-associated GABAergic remodeling along with the failure of anti-glutamatergic and acetylcholinesterase inhibitor therapies to halt the progression of the disease could point to the GABAergic system as a promising therapeutic target for AD. In this review, we have presented a large body of evidence potentially supporting this assertion. Further research will be important to shed light on the implications of GABAergic alterations in the disease, in order to assess the potential of GABAergic modulation to slow or stop disease progression and protect or restore cognition in AD patients.

## Figures and Tables

**Table 1:** Changes in GABA levels in the human Alzheimer’s disease brain.

Region	Change	References
Hippocampus	↓	[172,174,177]
	↔	[173,178,180]
Subiculum	↔	[174]
Thalamus (Whole/Subregion Unspecified)	↔	[175]
Thalamus (Dorsolateral Nucleus)	↔	[178]
Thalamus (Dorsomedial Nucleus)	↓	[173]
Thalamus (Ventrolateral Nucleus)	↓	[178]
	↔	[173,174]
Thalamus (Anterior Nucleus)	↔	[173,174]
Subthalamic Nucleus	↔	[174]
Cingulate Cortex	↓	[173,178]
	↔	[170,174]
Amygdala	↓	[172,178]
	↔	[174]
Caudate Nucleus	↔	[173,174,175]
	↑	[178]
Putamen	↔	[173,174,178]
Substantia Nigra	↔	[174,177,178]
Substantia Innominata	↔	[174,177]
Globus Pallidus (Whole)	↔	[174]
Globus Pallidus (Interna)	↔	[178]
Globus Pallidus (Externa)	↔	[178]
Nucleus Accumbens	↔	[173,174,178]
Septal Nuclei/Medial Olfactory Area	↔	[174]
Frontal Cortex (Subregion Unspecified)	↓	[177]
Frontal Cortex (Prefrontal Cortex)	↓	[173,174]
	↔	[172,179]
	↑↓	[170]
Frontal Cortex (Superior)	↔	[178]
Frontal Cortex (Orbitofrontal Cortex)	↓	[178]
	↔	[174]
Frontal Cortex (Premotor Cortex)	↓	[173]
	↔	[174]
Frontal Cortex (Primary Motor Cortex)	↓	[174]
Insular Cortex	↔	[178]
Temporal Cortex (Subregion Unspecified)	↓	[176]
Temporal Cortex (Superior)	↓	[170,174,175]
Temporal Cortex (Middle)	↓	[170,172,173,174]
Temporal Cortex (Inferior)	↓	[171,174]
	↔	[173,178]
Temporal Cortex (Temporal Pole)	↓	[173]
	↔	[174]
Temporal Cortex (Entorhinal Cortex)	↓	[173]
Temporoparietal Cortex (Posterior)	↓	[169]
Parietal Cortex (Superior)	↓	[170]
Parietal Cortex (Primary Somatosensory Cortex)	↓	[173]
Parietal Cortex (Somatosensory Association Cortex)	↔	[172,174]
Parietal Cortex (Angular Cortex)	↔	[178]
Occipital Cortex	↓	[177,178]
Occipital Cortex (Visual Cortex)	↓	[173,175]
	↔	[172]
Cerebellum	↓	[175]
	↔	[174]
Hypothalamus	↔	[174,178]
Hypothalamus (Mammillary Body)	↔	[178]
Nucleus Rubor	↔	[174]
Periaqueductal Gray	↔	[174]
Raphe Nucleus	↔	[178]
Pons (Basilar)	↔	[174]
Medulla Oblongata (Olivary Body)	↔	[174]

Changes were considered significant at *p* < 0.05. ↑ Increase, ↔ No change, ↓ Decrease, ↑↓ Increase or decrease depending on conditions/procedures.

**Table 2:** Alterations in GABAergic signaling component expression in the human Alzheimer’s disease brain.

Component	Methodology	Subregion	Change	Comments	References
Hippocampal Formation					
GABA_A_R α_1_	IHC	CA1, CA2, prosubiculum	↓		[235]
		DG, subiculum, presubiculum	↔		
	WB	CA1, CA2, CA3, DG, subiculum	↔		[236]
	ISH	CA1/subiculum, CA2, CA3, CA4, DG	↓		[237]
GABA_A_R α_5_	WB	CA1, CA2, CA3	↓	Significant change from mild/moderate to severe AD	[236]
		DG, subiculum	↔		
	ISH	CA1/subiculum, CA2, CA3, CA4, DG	↓		[237]
	Autoradiography	CA1	↓		[238]
		CA2, CA3, DG, subiculum, presubiculum, parasubiculum	↔		
GABA_A_R β_1_	WB	CA1, CA2, CA3, DG, subiculum	↔		[236]
GABA_A_R β_2_Subunit	WB	CA1, CA2, CA3, DG, subiculum	↔		[236]
	ISH	CA1, CA2, CA3, CA4, DG	↔		[240]
GABA_A_R β_3_ Subunit	ISH	CA1, CA2, CA3, DG	↓		[240]
		CA4	↔		
GABA_A_R β_2/3_ Subunit	IHC	CA1, CA2, CA3, subiculum	↔	Antibody specific for both β_2_ and β_3_	[239]
GABA_A_R γ_2_ Subunit	IHC	CA1, CA2/3, CA4, DG	↔	Qualitative study	[241]
GABA_A_R γ_1/3_ Subunit	IHC	CA1, CA2/3, CA4/DG	↑	Antibody specific for both γ_1_ and γ_3_, Qualitative study	[241]
GABA_B_R R1 Subunit	IHC	DG, CA2/3, CA4	↑↓	Antibody primarily recognizes R1a isoform, increase observed from control to moderate AD, but no change from control to severe AD—transient upregulation	[234]
		CA1, DG, subiculum	↔	Non-significant but trend as in other regions	
GAD65/2	IHC	DG	↓		[215]
GAT1	[^3^H]tiagabine radioligand binding	DG	↔		[244]
	IHC	CA1, CA2, CA3, DG, subiculum	↔		[218]
GAT3	IHC	DG	↑	Significant increase in reactive astrocytes	[181]
		CA1, CA3	↓	Observed in stratum pyramidale and also in astrocytes specifically	[218]
		Subiculum		Observed across region and in astrocytes specifically	
		CA2, DG	↔	Large, non-significant downregulation observed	
BGT1	IHC	CA2, CA3	↑	Observed in stratum radiatum and also in CA3 astrocytes specifically	[218]
		DG		Observed in all layers	
		CA1, subiculum	↔	Increase observed in astrocytes specifically	
**Cerebral Cortex**					
GABA_A_R	Autoradiography [^3^H] GABA radioligand binding	Superior frontal gyrus	↔	GABA_B_R blocked with baclofen	[233]
GABA_A_R α_1_ Subunit	qPCR	Prefrontal cortex	↓		[242]
		Temporal cortex			[243]
	WB	Temporal cortex	↓		[243]
GABA_A_R α_2_ Subunit	qPCR	Prefrontal cortex	↓	No change when patients classified by Aβ plaque load	[242]
		Temporal cortex			[243]
GABA_A_R α_4_ Subunit	qPCR	Prefrontal cortex	↓		[242]
GABA_A_R α_5_ Subunit	Autoradiography	Entorhinal cortex, perirhinal cortex	↓		[238]
		Temporal cortex	↔		
	qPCR	Temporal cortex	↓		[243]
GABA_A_R β_1_ Subunit	qPCR	Temporal cortex	↔		[243]
	WB				
GABA_A_R β_2_ Subunit	qPCR	Temporal cortex	↓		[243]
GABA_A_R β_3_ Subunit	qPCR	Temporal cortex	↓		[243]
GABA_A_R γ_1_ Subunit	qPCR	Temporal cortex	↔		[243]
	WB				
GABA_A_R γ_2_ Subunit	qPCR	Prefrontal cortex	↔		[242]
		Temporal cortex	↓		[243]
	WB	Temporal cortex	↓		[243]
GABA_A_R δ Subunit	qPCR	Prefrontal cortex	↓		[242]
		Temporal cortex			[243]
GABA_A_R ε Subunit	qPCR	Prefrontal cortex	↔		[242]
GABA_A_R θ Subunit	qPCR	Prefrontal cortex	↔		[242]
GABA_B_R R1 Subunit	qPCR	Prefrontal cortex	↔		[242]
GABA_B_R R2 Subunit	qPCR	Prefrontal cortex	↓	Patients classified by Aβ plaque load	[242]
GABA_B_R	Autoradiography [^3^H]GABA radioligand binding	Superior frontal gyrus	↓	GABA_A_R blocked with isoguvacine	[233]
GAD67/1	qPCR	Prefrontal cortex	↓	Patients classified by Aβ plaque load	[242]
	WB	Middle temporal gyrus	↔	Only 2 AD and 2 control cases	[215]
GAD65/2	IHC	Middle temporal gyrus	↓	Layers II/III and IV	[215]
		Primary visual cortex	↔	Trend towards reduction	
	WB	Middle temporal gyrus	↓	Only 2 AD and 2 control cases	[215]
GATs	[^3^H]nipecotic acid radioligand binding	Temporal cortex	↓	Nipecotic acid is a GABA reuptake inhibitor	[219]
		Frontal cortex	↔		
GAT1	[^3^H]tiagabine radioligand binding	Frontal cortex	↔		[244]
		Temporal cortex			
	IHC	Superior temporal gyrus	↓	Observed across both regions, and also in astrocytes specifically	[218]
		Entorhinal cortex			
GAT3	IHC	Superior temporal gyrus	↔	Large, non-significant downregulation observed	[218]
		Entorhinal cortex	↓	Observed across region, and also in astrocytes specifically	
BGT1	IHC	Superior temporal gyrus	↑		[218]
		Entorhinal cortex	↔	Increase observed in astrocytes specifically	
**Subcortical Structures**					
GAD67/1	ISH	Hypothalamus (superchiasmatic nucleus and retrochiasmatic area)	↔		[214]
	ISH	Caudate nucleus, putamen	↑	Attributed to increase in neuron number, but no increase per neuron	[216]
		Ventral striatum	↔		
GAD65/2	ISH	Hypothalamus (superchiasmatic nucleus and retrochiasmatic area)	↔		[214]
	IHC	Putamen	↓		[215]
		Globus pallidus	↔		
GATs	[^3^H]nipecotic acid radioligand binding	Caudate nucleus, putamen, globus pallidus	↔	Nipecotic acid is a GABA reuptake inhibitor	[219]

↑ Increase, ↔ No change, ↓ Decrease, ↑↓ Increase or decrease depending on conditions/procedures. Abbreviations: γ-aminobutyric acid (GABA), GABA_A_ receptor (GABA_A_R), GABA_B_ receptor (GABA_B_R), dentate gyrus (DG), cornu ammonis (CA), glutamic acid decarboxylase (GAD), GABA transporter (GAT), immunohistochemistry (IHC), Western blot (WB), in situ hybridization (ISH), quantitative polymerase chain reaction (qPCR), Alzheimer’s disease (AD), amyloid-β (Aβ).
